# Functional expression of polyethylene terephthalate-degrading enzyme (PETase) in green microalgae

**DOI:** 10.1186/s12934-020-01355-8

**Published:** 2020-04-28

**Authors:** Ji Won Kim, Su-Bin Park, Quynh-Giao Tran, Dae-Hyun Cho, Dong-Yun Choi, Yong Jae Lee, Hee-Sik Kim

**Affiliations:** 1 Cell Factory Research Center, Korea Research Institute of Bioscience and Biotechnology (KRIBB), Daejeon, 34141 Republic of Korea; 2 Department of Environmental Biotechnology, KRIBB School of Biotechnology, University of Science & Technology (UST), Daejeon, 34113 Republic of Korea

**Keywords:** Polyethylene terephthalate (PET), PET hydrolase (PETase), Plastic degradation, Microalgae, *Chlamydomonas reinhardtii*, Bioremediation

## Abstract

**Background:**

For decades, plastic has been a valuable global product due to its convenience and low price. For example, polyethylene terephthalate (PET) was one of the most popular materials for disposable bottles due to its beneficial properties, namely impact resistance, high clarity, and light weight. Increasing demand of plastic resulted in indiscriminate disposal by consumers, causing severe accumulation of plastic wastes. Because of this, scientists have made great efforts to find a way to biologically treat plastic wastes. As a result, a novel plastic degradation enzyme, PETase, which can hydrolyze PET, was discovered in *Ideonella sakaiensis* 201-F6 in 2016.

**Results:**

A green algae, *Chlamydomonas reinhardtii*, which produces PETase, was developed for this study. Two representative strains (*C. reinhardtii* CC-124 and CC-503) were examined, and we found that CC-124 could express PETase well. To verify the catalytic activity of PETase produced by *C. reinhardtii*, cell lysate of the transformant and PET samples were co-incubated at 30 °C for up to 4 weeks. After incubation, terephthalic acid (TPA), i.e. the fully-degraded form of PET, was detected by high performance liquid chromatography analysis. Additionally, morphological changes, such as holes and dents on the surface of PET film, were observed using scanning electron microscopy.

**Conclusions:**

A PET hydrolyzing enzyme, PETase, was successfully expressed in *C. reinhardtii*, and its catalytic activity was demonstrated. To the best of our knowledge, this is the first case of PETase expression in green algae.

## Background

Plastic pollution is one of the most important environmental issues in the world. In 2014, it was estimated that up to 51 trillion pieces (236 kilotons) of microplastic float in the world’s oceans [1, 2] and that much larger amounts of plastic will continue to flow into the oceans [3, 4]. Recent meta-analysis studies have shown that polyethylene (PE), polyester and acrylics (PP&A), polypropylene (PP), and polystyrene (PS) are the most abundantly present plastics in aquatic systems [5, 6]. A major problem is that most plastics cannot be degraded naturally. Once plastics are discarded into the environment, they are broken into small-sized fragments by abiotic stresses, and are left to accumulate for millions of years [7, 8].

Polyethylene terephthalate (PET) is a very common plastic. It is mainly used to make beverage bottles because of its impact resistance, light weight, and high clarity [9]. Due to its outstanding mechanical properties and stability, PET consumption has increased for the decades. In 2019, the global PET market was 39.8 billion USD and it was expected to reach 51 billion in 2024 with a CAGR of about 4.2% [10]. According to the increase of consumption, PET waste has become a large portion of plastic pollution. In response, different strategies to treat or recycle the PET have been investigated. For example, mechanical processes for PET separation or chemical processes for PET decomposition were reported, and it led the increase of gross PET recycling rate [11–13]. However, only clean PET wastes without the residual foods or dregs are able to be treated by the processes, thus the recycling rate has been rather decreased in recent several years [14]. Currently, most of non-recyclable PET wastes are incinerated, then a large amount of air pollutant such as CO_2_ is emitted during this process [14–16]. Therefore, the biological treatments for plastic waste have been attracting attention. To date, scientists have verified 27 enzymes that degrade synthetic polymers or oligomers [17], and most of the enzymes were lipase, cutinase, and esterase [18, 19]. However, in many cases, plastic degradation is not occurred by single enzyme: multiple enzymes and metabolic pathways have to be utilized [14, 20–22].

In 2016, *Ideonella sakaiensis* 201-F6, which can degrade and use PET as a sole carbon source, was isolated from PET waste [23]. Researchers identified two novel enzymes from *I. sakaiensis* 201-F6: PET hydrolase (PETase) and mono(2-hydroxyethyl) terephthalic acid hydrolase (MHETase), both of which demonstrate PET-degrading activity. These enzymes hydrolyze PET into non-toxic monomers (e.g., terephthalic acid (TPA), ethylene glycol (EG)) [23, 24]. After the discovery of PETase, many studies have been performed in order to verify its structure and enhance its activity [24–27]. Additionally, production of PETase was studied in bacterial systems for its potential application in biological PET recycling [16, 28, 29]. Bacterial systems offer various advantages for the production of PETase: high growth rate, low cost, easiness of manipulation, well-established genetic tools, etc. However, bacteria may also be considered as a pollutant because of endotoxins or needing a rich carbon source for growth. Moreover, the rapid growth of bacteria is another threat if it flows into the environment.

Microalgae is much more suitable for environmental applications because they do not require organic carbon sources under photoautotrophic conditions and usually do not have endotoxins [30]. For these reasons, a diatom, *Phaeodactylum tricornutum* was utilized for the production of PETase recently. PETase was secreted by using *P. tricornutum* as a host, and enzymatic activity was demonstrated through the liquid chromatography and electron microscopy [31]. However, *P. tricornutum* requires low temperatures, silica as a nutrient, and high salinity to grow. These features limit the scope of application, so an alternative microalgal host is needed to be utilized for producing PETase.

*Chlamydomonas reinhardtii* is a unicellular, photosynthetic microorganism that has diverse advantages as a model organism [32–34]. Because *C. reinhardtii* is considered to be ‘generally recognized as safe (GRAS)’, it is suitable for environmentally-friendly applications. In this study, we focused on functional expression of PETase in *C. reinhardtii*. To generate a stable transformant, two *C. reinhardtii* strains were compared. After transformation, the expression of PETase was confirmed by western blotting. The activity of PETase was demonstrated quantitatively and qualitatively through the high performance liquid chromatography and scanning electron microscopy.

## Results

### Transformation of microalgae

**Fig. 1:**
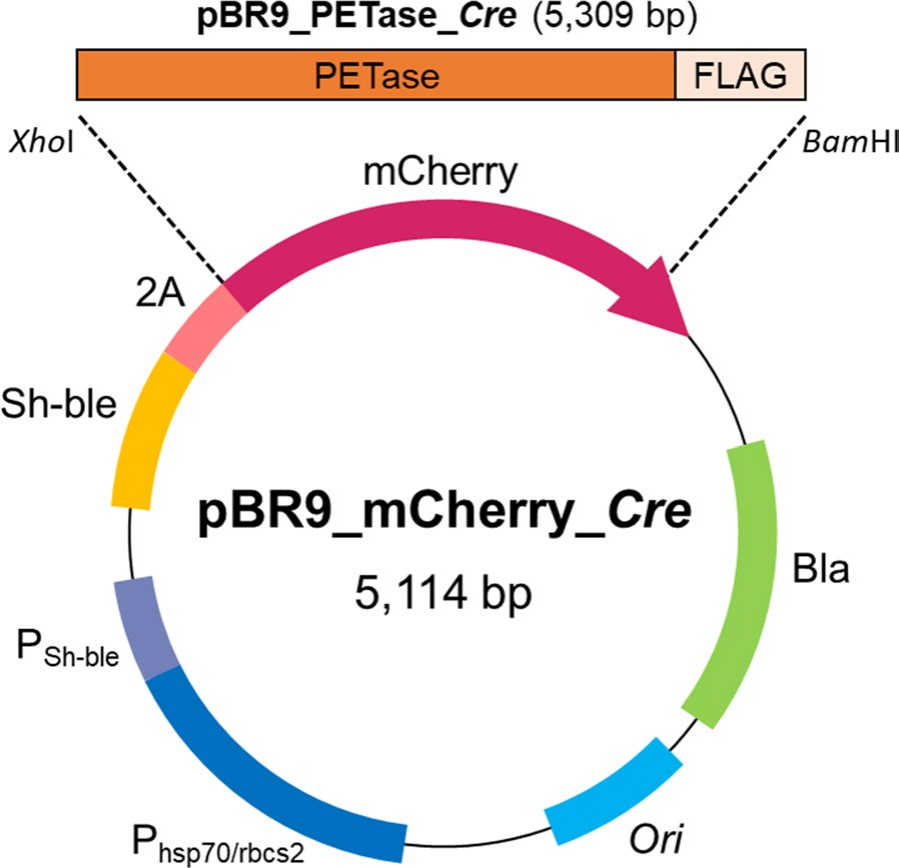
Structure of backbone plasmid and plasmid construction strategy. Bla and Sh-ble represent Ampicillin and Zeocin resistance gene, respectively. *Ori* represents origin of replication for *E. coli*

**Fig. 2:**
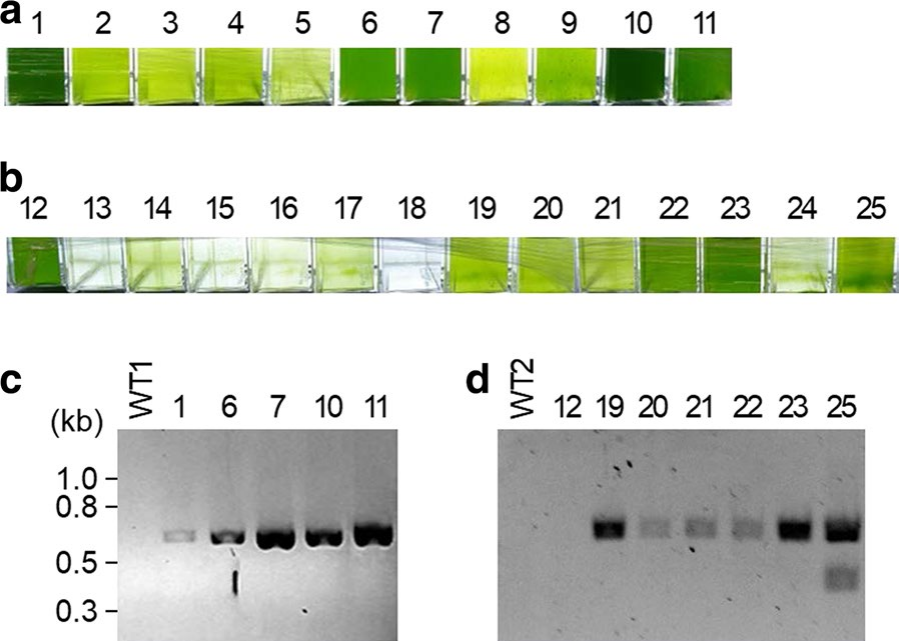
Selection of PETase transformants. **a**, **b** Final culture solution of CC-124 (**a**) and CC-503 (**b**) transformants. **c**, **d** PCR results of CC-124 (**c**) and CC-503 (**d**) transformants for confirming the gene integration. Numbers represent clone #. WT1 and WT2 represent CC-124 and CC-503 wild type, respectively

*Chlamydomonas reinhardtii* CC-124 (mt− [137c]) is a common laboratory wild-type strain, which carries the *nit*1 and *nit*2 mutations and is usually used for gene transformation. *C. reinhardtii* CC-503 (cw92 mt+) is a cell wall-less mutant of CC-125 developed for efficient transformation. In this study, we examined these two strains for transformation and expression of the PETase gene. Codon-optimized PETase gene was substituted for the mCherry gene of pBR9_mCherry_Cre (resulting in pBR9_PETase_Cre), which is a high-strength expression vector for *C. reinhardtii* with the Sh-Ble-2A fusion expression system (Fig. 1) [35, 36]. By using this plasmid (pBR9_PETase_Cre), two strains were transformed via electroporation. The cells were spread on an agar plate containing Zeocin. The antibiotic-resistant colonies were obtained from each plate, and then 288 colonies were inoculated and cultivated in 96-well plates. After cultivation in the 96-well plates, 61 clones of CC-124 and 17 clones of CC-503 were grown with TAP medium containing 10 mg/L Zeocin. The grown cells were transferred to 24-well plates, followed by 12-well plate cultivations. 11 clones of CC-124 and 14 clones of CC-503 were grown in 12-well plates, and then the clones were cultivated in 10 mL of media in a T-25 flask. 5 out of 11 CC-124 clones (#1, 6, 7, 10 and 11) were well-grown, while 7 out of 14 clones of CC-503 (#12, 19, 20, 21, 22, 23 and 25) grew well (Fig. 2a, b). To confirm the gene integration of well-grown *C. reinhardtii*, polymerase chain reaction (PCR) was carried out by using Cre_Sh-ble_PETase F and R primers. All of the selected clones of CC-124 transformants showed bands of the correct size (727-bp) (Fig. 2c). 6 out of 7 CC-503 clones showed the bands of the correct size (Fig. 2d). Although CC-503 clone #12 demonstrated growth under the Zeocin-supplemented condition, the 727-bp band was not detected, even in repeated experiments.

### Expression of PETase in the microalgae

**Fig. 3:**
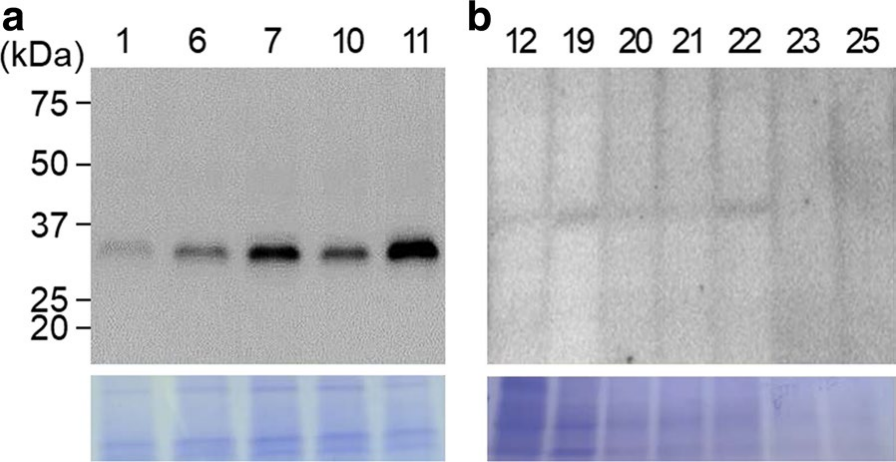
Expression of PETase in *C. reinhardtii*. **a**, **b** Western blot results of CC-124 (**a**) and CC-503 (**b**) transformants. Numbers represent clone #. Coomassie brilliant blue staining was performed for each western blot as a standard

To confirm the expression of PETase, western blot analysis was performed with the selected clones. For the CC-124 transformants, the cells were cultivated for 5 days and then used for protein sampling. All five of the clones expressed PETase (Fig. 3a). Clones #1 and #11 showed the lowest and highest expression level, respectively. The expression levels of each clone were retained consistently in repeated experiments. In contrast, all of the CC-503 transformants showed very slow and poor growth, thus requiring more than 2 weeks of cultivation to obtain the minimum amount of cells for protein sampling. In a western blot, faint bands appeared, but the size was incorrect. In addition, repeated experiments showed slightly different results, but no clear bands. Taking these results into consideration, we concluded that CC-124 is more appropriate for PETase expression. Clone #11 was selected as a final transformant and was used for the rest of the experiments.

### Validation of PETase activity against a PET bottle

**Fig. 4:**
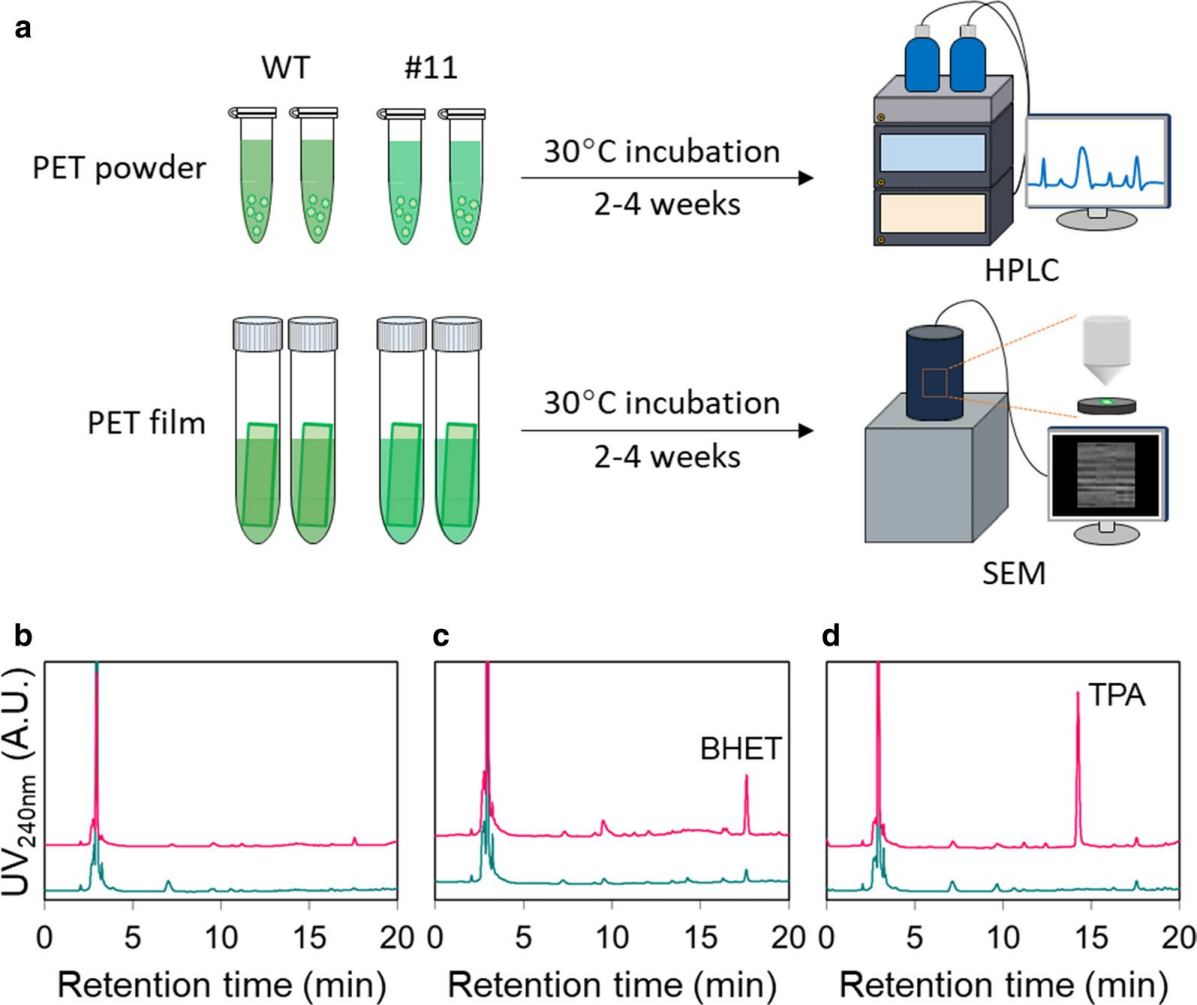
Catalytic activity assay of PETase produced by *C. reinhardtii*. **a** Schematic diagrams of the activity assay experiment strategies. **b**–**d** HPLC profiles of PETase powder incubation experiments: 2 weeks (**b**), 3 weeks (**c**) and 4 weeks (**d**) after incubation. Green and red lines indicate CC-124 wild type and CC-124_PETase #11 lysates, respectively

We have demonstrated that *C. reinhardtii* CC-124_PETase #11 expressed PETase at the highest level of the clones that were evaluated. To investigate whether the PETase produced by CC-124_PETase #11 exhibits activity against commercial PET bottles, two experiments were designed and performed (Fig. 4a).

For the first experiment, PET powder was prepared by scraping a commercial PET beverage bottle with sandpaper. The cell lysates of wild-type CC-124 and CC-124_PETase #11 supplemented with a protease inhibitor cocktail were prepared at 4 mg/mL of total protein. The PET powder and each lysate were combined in 1.5 mL screw-cap tubes, and allowed to incubate at 30 °C for 2 weeks. After incubation, supernatants of each lysate were collected and HPLC analysis was performed. The lysates of wild-type CC-124 and CC-124_PETase #11 showed no peaks of monomers such as MHET, BHET, or TPA (Fig. 4b). For further analysis, the same experiment was performed with 3 weeks of incubation. In the HPLC results, only the CC-124_PETase #11 lysate showed the peak of BHET, which is degraded form of PET (Fig. 4c). After 4 weeks, a TPA peak was observed in HPLC analysis of CC-124_PETase #11 lysate (Fig. 4d). For the quantitative analysis, the peak area of TPA was calculated. As a result, it was found that 9.12 mg of TPA was generated from 30 mg of PET powder. Considering stoichiometry, the conversion rate was 35.17%.

**Fig. 5:**
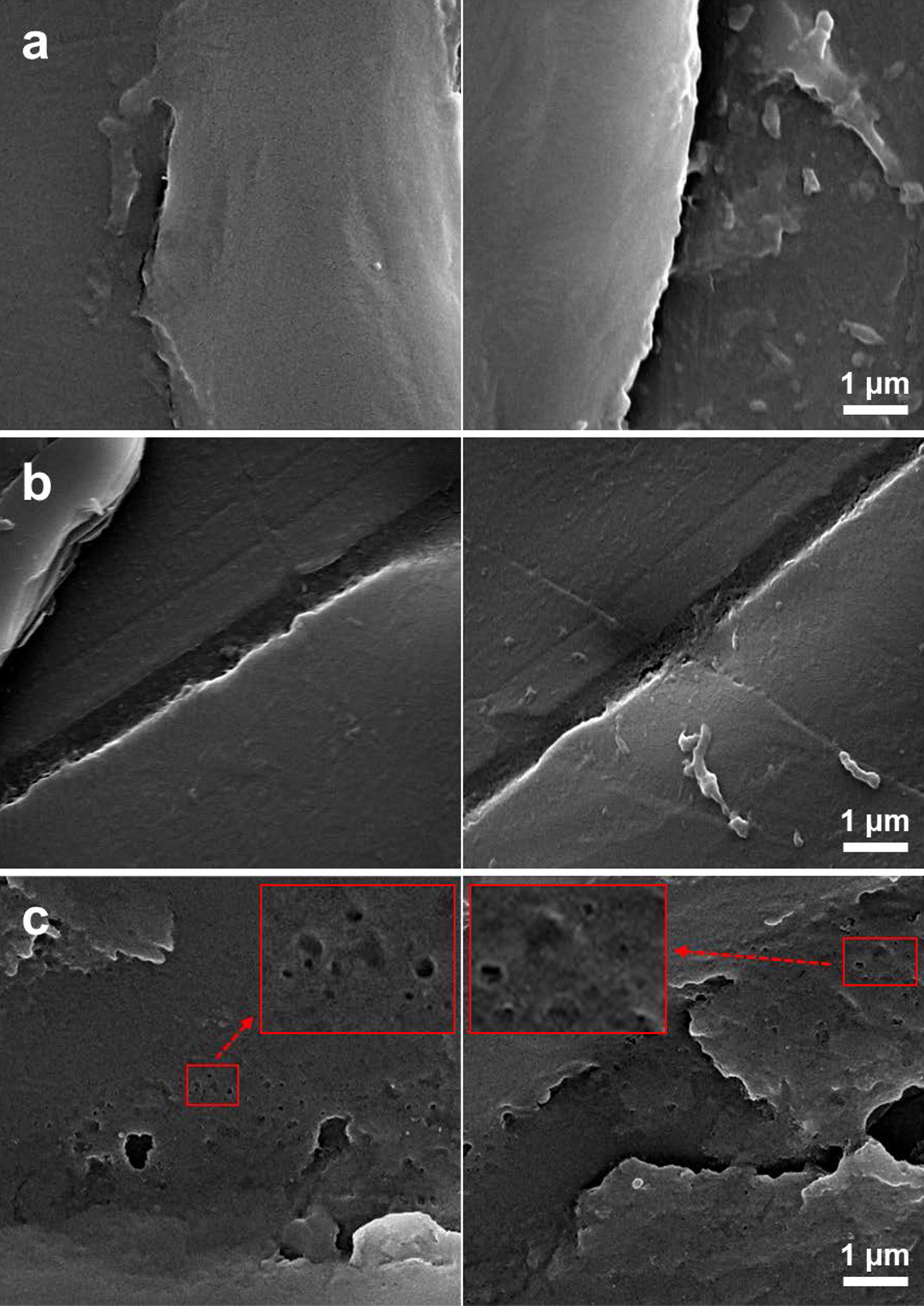
The results of scanning electron microscopy. **a**, **c** PET surface after 4 weeks incubation with CC-124 wild type (**a**) and CC-124_PETase #11 (**c**) lysate. **b** PET surface after 2 weeks incubation with CC-124_PETase #11. The magnitudes were ×20,000 for all samples. Small and large red boxes represent original and zoomed pictures, respectively

For the second experiment, 2 cm × 1 cm PET films were prepared and allowed to incubate at 30 °C with each cell lysate for 2 and 4 weeks. After incubation, the surfaces of the PET films were analyzed with scanning electron microscopy (SEM) to investigate the activity of PETase. Two weeks of incubation did not cause any structural changes on the surface of the PET film (Fig. 5b); this is in accordance with the results of the first experiment. In contrast, many holes and dents appeared after 4 weeks of incubation with CC-124_PETase #11 cell lysate (Fig. 5c), whereas wild-type cell lysate did not affect the surface structure of the film (Fig. 5a). The supernatant of 4th week CC-124_PETase #11 lysate, which was incubated with the PET film, was also analyzed using HPLC and a TPA peak was observed.

## Discussion

The discovery of PETase from *I. sakaiensis* was a vital breakthrough in plastic biodegradation research. Since then, numerous studies have been carried out and much progress has been made [27, 29, 37]. Structural and functional insights into plastic degrading enzymes obtained by current studies on PETase would be of great help in further research of other plastics (e.g., PP, PE, PS). However, despite these advances, little research has been done on the application of plastic degrading enzymes to the environment. Microalgae is an attractive host for plastic degrading enzyme production and environmental applications because they already live in almost every water system, usually do not produce endotoxins, and grow without needing organic carbon supplementation. In 2019, PETase production using the marine microalgae *P. tricornutum* was successfully demonstrated [31]. This report demonstrated the possibility of functional production of PETase in microalgae by using *P. tricornutum*, which is a representative model species of diatom. However, in general, *P. tricornutum* grows poorly compared to green algae species such as *Chlamydomonas* and *Chlorella* [38–40]. Thus, we chose *C. reinhardtii* as an alternative host, since the species grows quickly and is a freshwater microalga.

In the previous study, PETase produced from *P. tricornutum* showed a single peak of fully-degraded PET (TPA), after 10 days of culture [31]. The degradation was much faster than that of the *C. reinhardtii* derivatives used in this study, which only showed chemical and morphological changes after 4 weeks. In the *P. tricornutum* study, PETase was secreted into the culture medium. Therefore, the PETase would not be affected by endogenous proteases or other components, whereas in our study, the enzyme was exposed to these factors due to cell lysis. Despite of the slow reaction, we demonstrated that *C. reinhardtii*-derived PETase catalyzed PET with a high conversion rate. Considering that the inner side of PET bottle was coated and the bottle we used contained yellowish pigment, we believe that the conversion rate of about 35% was enough to address the potential. In addition, intracellular expression of PETase in *C. reinhardtii* may be beneficial for the specific application. For instance, PETase expressed in intact cells can be used to feed zooplanktons or fish to control the bioconcentration of microplastic. Also, in the *P. tricornutum* study, an activity-improved mutant, R280A, was used, so the degradation of PETase occurred much faster. If the R280A mutant is used for further studies, it will be helpful for enhancing the catalytic activity of PETase.

## Conclusions

In this study, we demonstrated the functional expression of PETase in a model green microalga, *C. reinhardtii*. Catalytic activity of PETase was proven by detecting TPA (i.e. the fully degraded form of PET) via HPLC analysis. Additionally, morphological changes on the surface of PET film after PETase treatment was observed by electron microscopy. These results suggest that microalgae are a potential biological treatment strategy for plastic pollution, especially in freshwater and terrestrial environments. To the best of our knowledge, this is the first reported success of PETase production in green microalgae. We believe that this study offers a standard for biological degradation of plastics by using green microalgae or other photosynthetic eukaryotes.

## Methods

### Plasmid construction, strains, and cell cultivation

All plasmids, oligonucleotide primers, and strains used in this study are listed in Table 1. The amino acid sequence of PETase (ISF6_4831) was obtained from UniProt (http://www.uniprot.org/). To obtain pIDT_PETase_Opt from a commercial service (Integrated DNA Technologies Inc. (IDT), Coralville, IA, USA), the amino acid sequence of PETase was reverse-translated, codon-optimized for *C. reinhardtii*, and synthesized by using the resulting pIDT_PETase_Opt. The codon-optimized PETase-encoding gene was digested and cloned into pBR9_mCherry_Cre (Chlamydomonas Resource Center (CRC), Saint Paul, MN, USA) by using *Xho*I and *Bam*HI restriction endonucleases, generating pBR9_PETase_Cre.

**Table 1:** Plasmids, oligonucleotides and strains used in this study

Name	Description or sequence (5′ to 3′)	Source
Plasmids		
pIDT_PETase_OPT	Commercially distributed plasmid including codon-optimized PETase gene for expression in *C. reinhardtii*	This study
pBR9_mCherry_Cre	Deposited plasmid, containing codon optimized mCherry-encoding gene	CRC
pBR9_PETase_Cre	pBR9 containing PETase-encoding gene for *C. reinhardtii* from pBR9_mCherry_Cre	This study
Oligonucleotides		
Cre_Sh-ble_PETase F	GCAACTGCGTGCACTTCGT	This study
Cre_Sh-ble_PETase R	TTCTCGCAAGCAAAGATCAGCGT	This study
Strains		
*E. coli* DH5α		RBC bioscience
*C. reinhardtii* CC-124	*C. reinhardtii* wild type strain carrying *nit*1 and *nit*2 mutation	CRC
*C. reinhardtii* CC-503	Cell-wall-less mutant originated from *C. reinhardtii* CC-125	CRC
*C. reinhardtii* CC-124 #1 to #11	*C. reinhardtii* CC-124 transformants harboring PETase expression cassette	This study
*C. reinhardtii* CC-503 #12 to #25	*C. reinhardtii* CC-503 transformants harboring PETase expression cassette	This study

In all of the gene cloning, maintenance, and amplification experiments, *Escherichia coli* DH5α was used and cultured at 37 °C with shaking (200 rpm). The culture medium was formulated using liquid LB broth (BD, Franklin Lakes, NJ, USA) supplemented with an appropriate concentration of antibiotics (100 mg/L ampicillin). For the transformation of PETase gene, expression confirmation, and plastic degradation, microalgal strains were used. *Chlamydomonas reinhardtii* CC-124 (mt− [137c]) and CC-503 (cw92 mt+) were purchased from CRC and cultured at 25 °C with shaking (120 rpm). For cell cultivation, light intensity was maintained at 100 μmol/m^2^/s and the culture medium was composed of tris–acetate-phosphate (TAP) medium [41] supplemented with antibiotics (10 mg/L Zeocin). Standard procedures were followed for all experiments including DNA manipulations and gene cloning [42].

### Transformation and clone selection

For gene cloning, the ligated plasmid (PETase gene from pIDT_PETase_Opt + pBR9_mCherry_Cre) was transformed into *E. coli* DH5α by using commercial heat shock competent cells (RBC Bioscience, New Taipei City, Taiwan). The heat-shocked cells were spread on LB-agar plates (BD) containing 100 mg/L Ampicillin and the correct clones were confirmed by polymerase chain reaction (PCR) and DNA sequencing. From the final *E. coli* clone, pBR9_PETase_Cre plasmid was purified from the 300 mL culture solution by using Nucleobond Xtra Midi Plus (Macherey–Nagel, Bethlehem, PA, USA) for the transformation of microalgae.

Competent cells of *C. reinhardtii* CC-124 and CC-503 were prepared by using MAX™ Efficiency Transformation Reagent for Algae (Invitrogen, Carlsbad, CA, USA). Each 400 µL of competent cells containing 10 µg of pBR9_PETase_Cre (linearized by *Psi*I) was subjected to electroporation by using 0.4 cm MicroPulser Electroporation Cuvettes (Bio-Rad, Hercules, CA, USA). The electroporation was performed with Gene Pulser Xcell Electroporation systems under the conditions of 500 V, 50 μF, and 800 Ω. After the pulse, 10 mL of TAP medium supplemented with 40 mM of sucrose was added to the cells immediately, and the cells were recovered for 16 h at 25 °C with shaking (80 rpm) under low light. The cells were plated on TAP-agar plates containing 10 mg/L Zeocin. Hundreds of colonies from the TAP-agar plates were inoculated into 96-well plates with 150 µL TAP medium containing 10 mg/L Zeocin. After cultivation, cells in green-colored wells were transferred to 24-well and 12-well plates sequentially using the aforementioned conditions. For the selection of stable transformants, the cells from 12-well plates were cultured with 10 mL TAP medium in a T-flask. The well-grown cells were chosen as the final clones. To verify the PETase gene integration into the nuclear genome of *C. reinhardtii*, the genomic DNA of the final clones was extracted via boiling with specific solution (1 M KCl, 10 mM EDTA, 100 mM of Tris–HCl, pH 9.5) for subsequent PCR analysis. For the PCR analysis, PETase_Sh-ble_Cre F and R primers were used (Table 1).

### SDS-PAGE and western blot analysis

To analyze protein expression, algal cells were harvested by centrifugation at 6000 rpm. To extract protein, the cells were resuspended in RIPA buffer supplemented with protease inhibitor cocktail (Sigma Aldrich, St. Louis, MO, USA) and disrupted by bead-beating with 0.1 mm zirconia/silica beads for 5 min. Protein concentration of lysates were determined using the Pierce BCA Protein Assay Kit (Thermo Fisher Scientific, Waltham, MA, USA). The samples (50 μg/sample) were separated on 4-20% Mini-PROTEAN TGX™ Precast Protein Gel (Bio-Rad) according to standard SDS-PAGE procedure [42]. The gels were transferred onto a PVDF membrane using a Trans-Blot Turbo Transfer System (Bio-Rad). After transfer, the membrane was incubated with blocking solution (5% skim milk solution with 1× TBS-T) at room temperature for an hour. The membrane was then incubated with blocking solution supplemented with 1:5000-diluted mouse anti-FLAG M2 monoclonal antibody (Sigma Aldrich) at room temperature for 1 h. After antibody binding, the membrane was washed with 1× TBS-T four times (20 min/wash). To visualize the bands, the membrane was treated with ECL™ Prime (GE Healthcare, IL, USA) and the chemiluminescent signal was detected using a FUSION Solo (Vilber Lourmat, Collegien, France) imaging system.

### Catalytic activity assay of PETase

To analyze the catalytic activity of PETase produced from *C. reinhardtii*, a commercial PET beverage bottle (PepsiCo., NY, USA) was used as the substrate. The PET powder for HPLC analysis was prepared by grinding the bottle with sandpaper. The ground PET was filtered using a sieve to remove large fragments. The PET films for electron microscopy were prepared by cutting a PET bottle into 2 cm × 1 cm fragments. Wild-type *C. reinhardtii* CC-124 and CC-124_PETase #11 were cultivated and harvested to obtain cell lysates. The harvested cells were resuspended in 1× PBS and Protease Inhibitor Cocktail (Sigma Aldrich). The solution was disrupted by sonication on ice for 20 min with 3 s pulses at 7 s intervals (VC750; Sonics & Materials, Newtown, CT, USA). After the preparation of all materials, PET powder and films were incubated with 1 mL and 5 mL of the cell lysates, respectively. All of the reaction mixtures were incubated at 30 °C for 4 weeks, changing the cell lysate to fresh one weekly to prevent contamination and PETase degradation. At each time point, the solutions were combined and subjected to HPLC analysis. HPLC analysis was performed using an HPLC System™ (Agilent Technologies, CA, USA) equipped with a diode array detector. A C18 column (ZORBAX RR Eclipse Plus C18, 95 Å, 4.6 × 150 mm, 3.5 µm; Agilent Technologies) was used and the signals were detected at 240 nm. The mobile phase was comprised of 50 mM phosphoric acid and methanol. Chromatography was performed using a methanol gradient (0 min 20% methanol, 2 min 20% methanol, 12 min 40% methanol, 20 min 20% methanol; flow rate of 0.5 mL/min) at 40 °C.

### Scanning electron microscopy analysis

After incubation with algal lysates, PET films were washed with distilled and deionized water and dried for an hour. The PET samples were cut into a compact size (< 25 mm^2^) and were attached to the top of aluminum specimen stubs. They were then sputtered with a piece of double-sided metal sticky tape and gold-coated under vacuum using a sputter coater (Q15ORS; Quorum, UK). The samples were analyzed using a scanning electron microscope (FEI Quanta 250 FEG, FEI) at 10 kV.

## Data Availability

All data generated or analyzed during this study are included in this published article.

## Abbreviations

PET: Polyethylene terephthalate
TPA: Terephthalic acid
EG: Ethylene glycol
MHET: Mono(2-hydroxyethyl) terephthalic acid
BHET: Bis(2-hydroxyethyl) terephthalic acid
HPLC: High performance liquid chromatography
SEM: Scanning electron microscopy
